# Genome Sequence of *Arthrobacter globiformis* B-2979 phage Ichiang

**DOI:** 10.17912/micropub.biology.001879

**Published:** 2025-12-08

**Authors:** Jazmine Aftabi, Shivani Gangireddy, Uttej Kollu, Charlie Lu, Calista K Ly, Luke A Metcalf, Eshaan Patnaik, Dhruv Reddy, Jacob Taylor, Harry Teng, William Turula, Nalani M Wooton, Annie Zhou, Nancy E Castro

**Affiliations:** 1 Biological Sciences, University of Southern California, Los Angeles, United States; 2 Chemistry, University of Southern California, Los Angeles, United States; 3 Neuroscience, University of Southern California, Los Angeles, United States

## Abstract

Bacteriophage Ichiang is a siphovirus capable of infecting *Arthrobacter globiformis* B2979. The genome has a length of 41,984 base pairs with 64.8 % GC content and contains 64 predicted protein-coding genes and 1 tRNA gene. Based on gene content, Ichiang has been assigned to Actinobacteriophage FF cluster.

**Figure 1. Characteristics of the Arthrobacter globiformis B-2979 phage Ichiang:**
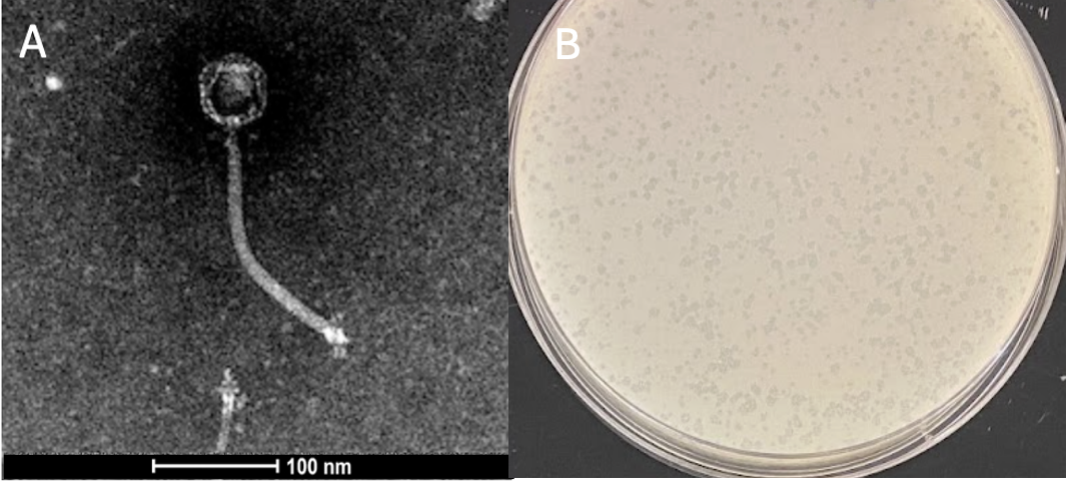
A) Negative-stain transmission electron micrograph revealed a siphovirus morphology. Scale bar is 100nm. B) Plaques of phage Ichiang. Plate is 100 mm in diameter.

## Description

Bacteriophages are viruses that infect and replicate in specific bacterial cells and are thus being developed for treating antibiotic-resistant bacterial infections (Hatfull, 2022). Here, we described the isolation and characterization of a novel bacteriophage, Ichiang.

Ichiang was isolated from a dry soil sample that was collected at the University of Southern California, GPS coordinates 34.02819 N, 118.28486 W. The soil sample was incubated with peptone-yeast extract-calcium (PYCa) medium while vigorously shaking for 1-2h. The sample was then centrifuged at 2,000 x g for 10 min to pellet the soil. The supernatant was filtered (0.22µm), then inoculated with *Arthrobacter* *globiformis* B-2979 and incubated while shaking at 30˚C for 48h (Zorawik et al., 2024). The resulting culture was refiltered, the filtrate plated in top agar with A. globiformis, and plates incubated at 30˚C for 24h. Ichiang produced small, clear, and uniform plaques with a diameter of approximately 1 mm (n=3) (Figure 1). The phage was purified through three rounds of picking from plaques and plating on lawns of A. globiformis, after which a lysate was prepared and used for negative-stain transmission electron microscopy. Ichiang has a siphovirus morphology with a capsid diameter of approximately 50-52 nm, and a tail length of approximately 200-203 nm (n=3) (Figure 1).

The phage DNA was extracted using the Promega Wizard DNA cleanup kit, prepared for sequencing using the NEB Ultra II Library kit, and sequenced on an Ilumina NextSeq 1000 (Russell and Hatfull, 2018). The resulting 2,380,586 100-base single end reads were trimmed with cutadapt 4.7 (using the option: –nextseq-trim 30) and filtered with skewer 0.2.2 (using the options: -q 20 -Q 30 -n -l 50) prior to assembly using Newbler v2.9 and checking for accuracy, completeness, and genomic termini using Consed v29 (Gordon, 2013). The resulting assembly had 5284x coverage for a genome 41,984 bp in length, with 64.8 % GC content and a 3' single-stranded overhang consisting of 12 bases (5' - TCCGCCGCGTGA - 3').

The genome was automatically annotated using DNA Master v5.23.6, containing programs Glimmer v3.02 (Delcher et al., 2007) and GeneMark v2.0 (Besemer and Borodovsky, 2005) for gene prediction. Starterator was used to refine gene start sites (http://phages.wustl.edu/starterator/). HHPRED (specific databases include: PDB mmCIF70, Pfam-A, and NCBI Conserved Domain databases (CD)_v3.19, and SMART_v6.0) (Söding et al., 2005), NCBI BLAST (Actinobacteriophage proteins, non-redundant protein sequences [nr]) (Altschul et al., 1990), and Phamerator (database Actino_Draft) (Cresawn et al., 2011) were used as comparative tools to identify the putative functions of proteins encoded by the predicted genes. Default parameters were used for all software programs, unless otherwise noted. Ichiang encodes 64 putative protein-coding genes, including 1 tRNA identified by Aragorn v1.2.38 (Laslett and Canback 2004) and tRNAscan-SE v2.0 (Lowe and Eddy 1997). Of these, 28 genes were assigned a putative function, while 36 genes could not be assigned a function. Ichiang was assigned to cluster FF based on gene content similarity of at least 35% to phages in the Actinobacteriophage database, phagesdb (https://phageDB.org) (Pope et al., 2017; Russell and Hatfull, 2017).

Two genes predicted to encode integrase functions were identified in the genome, suggesting the phage is able to establish lysogeny, as experimentally demonstrated for another cluster FF phage encoding both integrase homologues (Bethany & Sivanathan, 2025). The genome organization is very similar throughout the FF cluster, where the first third of the genome encodes functions related to virion structure and assembly, the central genes encoding a lysogeny cassette, and the remaining genes encoding proteins involved in DNA metabolism. All but 8 genes located within the central region of the genome are transcribed unidirectionally.

Nucleotide sequence accession number for Ichiang is available at GenBank with Accession No. PX307267 and Sequence Read Archive (SRA) No. SRX28943169.
